# Human-Elephant Conflicts and Villagers’ Attitudes and Knowledge in the Xishuangbanna Nature Reserve, China

**DOI:** 10.3390/ijerph17238910

**Published:** 2020-11-30

**Authors:** Kaiwen Su, Jie Ren, Jie Yang, Yilei Hou, Yali Wen

**Affiliations:** School of Economics and Management, Beijing Forestry University,35 Qinghua East Road, Beijing 100083, China; sukaiwen@bjfu.edu.cn (K.S.); renjie@bjfu.edu.cn (J.R.); yangjie2020@bjfu.edu.cn (J.Y.)

**Keywords:** Asian elephant, human–elephant conflict, Xishuangbanna, wildlife damage

## Abstract

In this study, we analyzed the accidents associated with the Asian elephant (*Elephas maximus Linnaeus*) and issues pertaining to compensation in Xishuangbanna Nature Reserve from 2011 to 2018. We conducted interviews and a questionnaire survey with 217 villagers. The results show that: (1) the main Asian elephants damage is crop loss (more than 95% of the total damage), and the villagers suffer economic losses; (2) through the influence of traditional culture and natural education, the majority of local villagers still have a favorable impression of Asian elephants; (3) female respondents, those engaged in agricultural production, those who had experienced crop loss and those who had never seen Asian elephants had more negative attitudes toward Asian elephants; (4) most villagers believe that the Asian elephant population has increased in the past decade; and (5) the villagers are quite passive in the human–elephant conflict, and most of them do not take action. Finally, based on the research results, this paper discusses the causes of human elephant conflict and proposes targeted mitigation measures.

## 1. Introduction

Rapid economic and population growth impose huge demands and pressure on wildlife resources and their living environment. Human–wildlife conflict becomes inevitable when the area for human settlement overlaps with wildlife habitat [1]. Human activities are increasingly affecting every ecosystem on Earth [2]. This leads to a decline in wildlife habitat quality and a decrease in natural food sources available to animals, and aggravates the conflict between humans and wildlife [3,4,5]. Wildlife accidents lead to the deterioration of the relationship between humans and wildlife, and hunting threatens the existence of wild animals [6,7]. Dealing with the relationship between human beings and wildlife and establishing a coexistence mechanism between them are challenging tasks [8,9,10].

Human beings have a responsibility to protect endangered wildlife. However, when the wildlife population recovers, the habitats that are situated close to or even overlap with the human living space may not be able to fully support their survival, which leads to new problems. When the needs and behaviors of wildlife have a negative impact on human goals and interests, or the goals and interests of human have a negative impact on the needs and behaviors of wildlife, human–wildlife conflicts will develop, a common example is wildlife damage [11,12].

Wildlife damage can be divided into three categories: (1) the damage caused by wild herbivores such as elephants, wild boars, and birds, which mainly rely on crops as food sources, causing economic losses to human beings [13,14,15]; (2) the damage caused by wild large carnivores such as tigers, bears, wolves, and leopards, which hunt poultry and other livestock [16,17,18]; and (3) wildlife damage to human life caused mainly by large carnivores and poisonous snakes [9,19,20]. Many studies have found that the frequency of conflicts between people and wildlife and economic losses are increasing in many areas [21,22,23,24].

In China, the human–elephant conflict is the most prominent of all wildlife destruction. Wild Asian elephants (*Elephas maximus Linnaeus*) cause injuries and damage to crops every year [19,25]. The Asian elephant is one of China’s Class 1 protected animals, and it is listed as an endangered species by the International Union for the Conservation of Nature and Natural Resources. Three thousand years ago, Asian elephants were widely distributed in China. Traces of Asian elephants can be found in most areas south of the Yellow River Basin [26]. The distribution of Asian elephants has decreased sharply with the development of human civilization. At present, the population of Asian elephants in China is estimated to be 216–243 [27], most of which are distributed in Xishuangbanna [28], except for the sporadic distribution in Pu’er City and Nangunhe National Nature Reserve [29].

In a period of 40 years, the Asian elephant population in Xishuangbanna has substantially increased, from 101 in 1976 to about 184–205 in 2016 [27]. However, extensive deforestation has resulted in habitat loss and fragmentation of Asian elephants [30,31]. Human settlements and plantations have replaced forests in many areas [32]. The traditional migration corridor of the Asian elephant has been cut off [33], and the area for human settlement and the distribution area for the Asian elephant gradually overlap. As a result, accidents associated with the Asian elephant have become a serious local social problem. At the same time, the human–elephant conflict not only causes economic losses, but also affects and threatens the daily activities and safety of local residents. Once the conflict exceeds the tolerance of local villagers, it is likely to endanger the villagers’ enthusiasm for wildlife conservation. [34].

There is an urgent need to protect and manage Asian elephants and alleviate the human–elephant conflict. Considering that the attitude and behavior of indigenous people directly affect the effective implementation of conservation policies and the scientific construction of coexistence mechanisms for human beings and wildlife [35], it is necessary to conduct research on the relationship between humans and elephants. This study has examined the following: (1) the current status of the human–elephant conflict in Xishuangbanna National Nature Reserve, (2) the attitudes and knowledge of villagers regarding Asian elephants, (3) the causes of human–elephant conflict, and (4) targeted mitigation countermeasures were put forward to provide scientific support for alleviating the human–elephant conflict.

## 2. Materials and Methods 

### 2.1. Study Area

**Figure 1:**
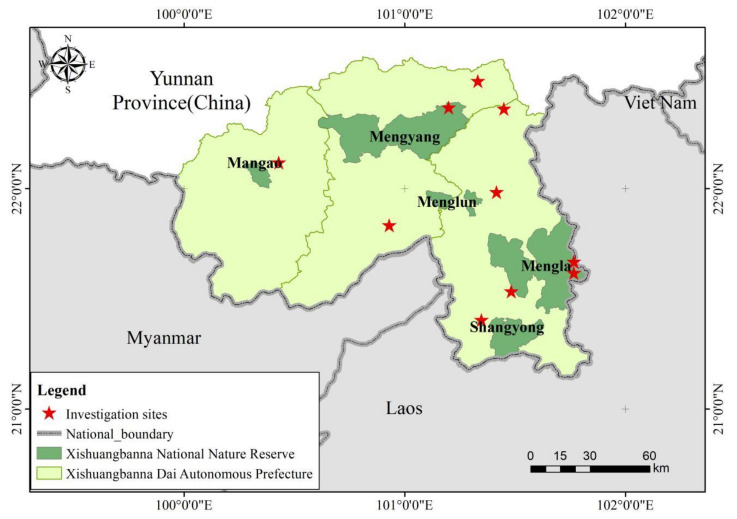
Study area.

Xishuangbanna National Nature Reserve is in Xishuangbanna Dai Autonomous Prefecture, Yunnan Province, China (N21°10’–22°14’, E100°16’–101°15’). It is composed of five subreserves, Mengyang, Menglun, Mengla, Shangyong, and Mangao, which are not connected to each other (Figure 1). The subreserves are located in 22 townships (towns) of Menghai, Mengla, and Jinghong. The total area of the nature reserve is 2474.39 km^2^, accounting for 12.68% of the land area of Xishuangbanna. There are 299 villages in and around the nature reserve, including 122 villages within the nature reserve and 177 villages around. The main protected objects of the nature reserve are the tropical forest marked by the rain forest on the northern edge of the tropics and the seasonal rain forest, the evergreen broad-leaved forest in the south subtropics dominated by the monsoon evergreen broad-leaved forest, and the rare and endangered wild animal and plant populations and their living environment in the tropics and south subtropics. There are abundant animal and plant resources in the nature reserve, including tropical rain forest, tropical seasonal rain forest, subtropical evergreen broad-leaved forest, deciduous broad-leaved forest, warm coniferous forest, bamboo forest, shrub, and grass. There are 727 species of vertebrates recorded, accounting for 1/5 of the total number of species in China (3317 species) and 1/3 of the Yunnan Province (1836 species) [36]. There are mainly rare and endangered endemic animals represented by Asian elephant, Indochinese tiger (*Panthera tigris* ssp. *corbetti*), water deer (*Rusa unicolor*), black bear (*Ursus thibetanus*), clouded leopard (*Neofelis nebulosa*), leopard (*Panthera pardus*), Indian bison (*Bos frontalis*), rhesus (*Macaca mulatta*), gray langur (*T. phayrei crepusculus*), green peafowl (*Pavo muticus*), and hornbill (*Anthracoceros coronatus*).

### 2.2. Data Collection

Since 2011, the Xishuangbanna government has cooperated with the Xishuangbanna Central Branch of China Pacific Insurance Company (CPIC) to sign a wildlife public liability insurance contract. All villagers have the right to report losses and receive compensation when they are affected. Government departments are responsible for insurance, and insurance companies are responsible for wildlife damage compensation. The data collection method involved field inspections conducted by the claim adjuster of the Yunnan branch of the CPIC, the Forestry and Grassland Bureau, the forestry station, the management and Protection Bureau of nature reserves, and the forest rangers to measure or estimate the quantity and loss of damaged crops.

In the compensation standard, crops were calculated in mu (1 mu = 1/15 ha) and cash crops were calculated by plant. The compensation limit for housing and ancillary facilities is 10,000 yuan, for death and disability is 200,000 yuan, and for medical expenses is 100,000 yuan. The compensation amount is adjusted with the fluctuation and change of the market economy, as shown in Table 1.

### 2.3. Methods

In May 2019, a presurvey was conducted to collect and record the historical situation and reality related to the local human–elephant conflict by interviewing the managers of the Natural Protection Department of Yunnan Forestry and Grassland Bureau, the Scientific Research Institute of Xishuangbanna Nature Reserve, the Forestry and Grassland Bureau of Xishuangbanna, the Asian elephant breeding center in Xishuangbanna, and the village heads of three villages bordering nature reserve status. Based on the integration of the opinions of many parties, the villages for formal investigation were selected, and 10 towns were selected for formal investigation. At the same time, based on the suggestions of local managers, the questionnaire was modified and improved.

We started a formal investigation in September 2019, and used semistructured interviews and a questionnaire survey as the main methods. The semistructured interview form provided an overall framework, and the questions were intentionally open to provide space for the respondents’ personal interpretation of the human–elephant conflict. This allowed respondents to express answers of uncertainty, complexity, and ambiguity [37,38]. This was quite important in view of the complexity of the social relations, natural environment, and stakeholders involved in the human–elephant conflict. Managers from the Scientific Research Institute of Xishuangbanna Nature Reserve, community work office of Xishuangbanna Nature Reserve, three management and protection stations (Mengla, Menghai, and Shangyong), the Reserve Management section of Xishuangbanna Forestry and Grassland Bureau, CPIC, and village heads of six villages seriously affected by human–elephant conflict were selected as the respondents of these semistructured interviews (S1). Twenty-two key informants were interviewed, including 14 males and 8 females. These key informants are the first-line participants in the management and protection of Asian elephants, and active participants in coordinating the relationship between humans and elephants. They can provide rich explanations on the causes, current situation, history, and villagers’ attitudes and behavior in relation to the conflict between humans and elephants. With the permission of the participants, most of the interviews were recorded. If this is not possible, comprehensive notes were taken.

The purpose of the interviews was to summarize the causes of the human–elephant conflict and the challenges in the process of alleviating the conflict. Respondents were first asked about their perceptions of human–elephant conflict based on their own experience. To elicit their initial ideas, the interviewees were first asked, “What is the relationship between the local people and Asian elephants?”. The interviewees were also asked about the factors used to assess the human elephant conflict in order to understand their objectivity in the process of evaluating the conflicts as well as how they dealt with the conflict between humans and elephants. They were asked to describe the main objects and scope of the accidents caused by the Asian elephants so as to determine the characteristics of the accidents and evaluate the compensation. Finally, the interviewees were asked about the causes of the conflict, the natural resources of the Asian elephant’s range of activities, and the countermeasures taken by different departments.

To accurately obtain the villagers’ attitudes and knowledge, this study adopted the questionnaire survey method. The questionnaire (S2) was improved based on the presurvey and semistructured interviews with managers, and the options that were not in line with local conditions were deleted and modified. In October 2019, 7 trained investigators conducted the questionnaire survey with 217 residents in 10 townships within and around the Xishuangbanna Nature Reserve (Table 2). To ensure the quality of the questionnaire, one-to-one interviews were conducted. The local area is inhabited by ethnic minorities and some people do not fluently speak the official language. To avoid communication barriers, three patrolmen from the Xishuangbanna Nature Reserve undertook the translation work. The information obtained through the questionnaire survey was divided into five parts: (1) the basic personal information of the interviewees, which included gender, age, occupation, education level, income source, etc.; (2) the effect of elephant conflict on the families of the interviewees, including personal safety, crop loss, and property loss; (3) the attitude and understanding of the interviewees towards Asian elephants and further inquiry into the reasons for their attitudes; (4) the population change of the Asian elephant in the last 10 years; and (5) the measures taken by the interviewees’ families in the face of human image conflict. Some participants checked the transcripts of the interviews to ensure the accuracy and validity of the data.

## 3. Results

### 3.1. The Present Situation of the Human–Elephant Conflict

#### 3.1.1. Types of Human–Elephant Conflict

In the interviews, the managers of Xishuangbanna Nature Reserve and Xishuangbanna Forestry and Grassland Bureau revealed that the Asian elephant damage in Xishuangbanna Nature Reserve and surrounding villages is mainly the loss of crops, including cash crops. The Asian elephants rarely attack humans or livestock. The human elephant conflicts occur mainly in mountainous areas, mid mountain areas, and nature reserves. The livestock that are mainly affected include cattle, pigs, sheep, etc., while the food crops include rice, corn, soybean, peanut, and sugarcane, and the cash crops include rubber, tea, coffee, and banana. Xishuangbanna has relatively benign natural conditions. Agricultural production can be carried out throughout the year, and crops are planted in all seasons. Therefore, the accidents associated with the Asian elephant are experienced throughout the year. However, the accidents frequently happen in April to November when crops are sown and mature. This is the period of greatest availability of crops that the elephants want to feed on, and in order to manage farmland, there more people present in the fields, increasing the probability of encountering Asian elephants.

Among the 217 households surveyed, 131 households suffered losses of food crops and cash crops in 2018, 14 households had their livestock attacked, and five households had been attacked by Asian elephants in 10 years. Chen Wenhui’s research in 2017 on Asian elephant accidents in China shows the same results [39]. In 2011, the total loss associated with Asian elephants in Xishuangbanna was 8.82 million yuan, accounting for 88.33% of all the losses caused by wildlife. Among them, there were three casualties, one cow was attacked, 9228.14 mu of food crops, 385,328 plants of cash crops, and other losses. Overall, the proportion of casualties and livestock loss was low, accounting for 2.92% and 0.03% of the total loss respectively. The loss of food crop and cash crop accounted for 48.90% and 46.15% of the total loss respectively, which together exceeded 95% of the total loss.

#### 3.1.2. Asian Elephants Damage 

**Figure 2:**
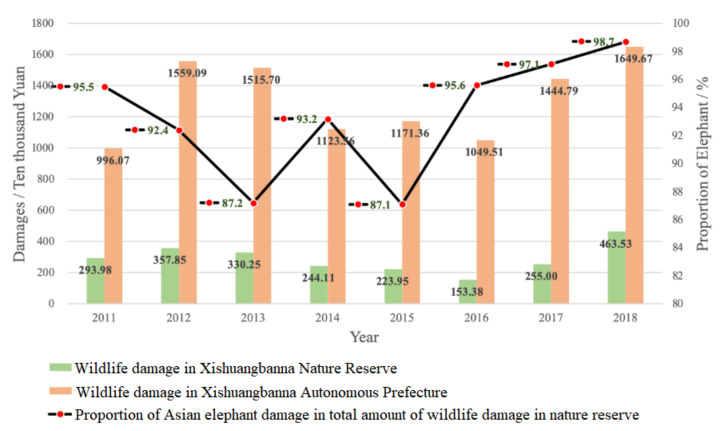
Wildlife damage in Xishuangbanna Nature Reserve from 2011 to 2018.

In terms of the number of villages in and around the nature reserve damaged by wildlife accidents from 2011 to 2018, the lowest number was 84 in 2011 and the highest was 232 in 2013. When the number of affected villages is the largest, they account for 77.6% of the total number of villages. The economic losses associated with wildlife accidents mainly include food, cash crops, property losses, and personal injuries. In 2011–2018, a total of 14,340 households in and around the nature reserve were damaged by wildlife, with a total loss of 23,220,497 yuan, accounting for 22.1% of the total amount of wildlife damage in Xishuangbanna Autonomous Prefecture, including 13,712,117 yuan for destroyed food crops, 91,566,668 yuan for economic crops, 72,850 yuan for animal husbandry, 83,025 yuan for property loss, and 736,737 yuan for other losses (including five injured and three dead). The Asian elephant damage is the biggest for all wildlife losses in the Xishuangbanna Nature Reserve. From 2011 to 2018, the total amount of Asian elephants damage accounted for 93.4% of the total amount of wildlife accidents in the reserve (Figure 2).

#### 3.1.3. Compensation for Accidents Associated with the Asian Elephants

Since 1991, the Xishuangbanna autonomous prefecture has made numerous compensations for wildlife accidents, which can be divided into three stages. The first stage is from 1991 to 2005. During this period, the compensation funds were jointly raised by the autonomous prefecture, provincial, and county governments, but the compensation funds were little, and the compensation standard was low. The second period is from 2006 to 2009. In 2005, the provincial government gave Xishuangbanna a one-time subsidy of 4 million yuan, and since 2006, the central government has provided a special subsidy of 5 million yuan per year. Governments at all levels have also increased investment in wildlife accident compensation funds. However, the compensation fund only accounts for 7% of the direct economic loss caused by wildlife in a particular year, and the compensation standard remains low. The third stage is from 2010 to the present. In 2010, Xishuangbanna began conducting the pilot work of public liability insurance for wild animals. The Xishuangbanna autonomous prefecture government cooperated with the CPIC to sign a public liability insurance contract for wild animals. The insurance company was responsible for the settlement of domestic and wildlife accidents, and the forestry department cooperated to complete the on-site investigation and loss determination and evaluation work, significantly improving the compensation standard (Table 3). From 2011 to 2018, the maximum compensation amount of Xishuangbanna Nature Reserve was 4.64 million yuan, and the lowest was 1.54 million yuan. The annual compensation amount accounts for 21.88% of the compensation amount of the entire autonomous prefecture.

### 3.2. Villagers’ Attitudes and Knowledge

#### 3.2.1. Villagers’ Attitudes toward Asian Elephants

The villagers expressed their attitudes toward Asian elephants. Most of them had a favorable attitude towards Asian elephants (*n* = 139, 64.1%), and did not want to see fewer Asian elephants. A small number of farmers had a dislike for Asian elephants (*n* = 78, 35.9%) and hoped that the number of Asian elephants would decrease. The main reason why some villagers liked Asian elephants is that the Asian elephants carry a symbolic value to the villagers (*n* = 61, 43.8%); they represent the local environment and culture. At the same time, Asian elephants are protected animals (*n* = 31, 22.3%). In addition, the villagers’ attitudes were influenced by Southern Buddhism (*n* = 47, 33.8%). The reason why villagers disliked Asian elephants was that the elephants are dangerous animals and endangered the lives and property of local residents (*n* = 70, 89.7%). In addition, Asian elephants are huge and frightening (*n* = 8, 10.2%).

In SPSS 22, the χ^2^ test was used to test the differences in villagers’ attitudes towards elephants in different demographic categories. The results showed that gender (χ^2^ = 7.246, *p* = 0.007), profession (χ^2^ = 7.343, *p* = 0.007), the crops that had been eaten by elephants (χ^2^ = 21.182, *p* = 0.000), and seen elephants (χ^2^ = 4.847, *p* = 0.028) had significant effects on the villagers’ attitudes towards elephants (Table 4). Male respondents had more negative attitudes towards Asian elephants than females. Respondents engaged in agricultural production were more negative than those in other industries. The villagers who had experienced the destruction of crops by the Asian elephants showed lower tolerance and a more negative attitude towards the Asian elephants. The villagers who had not experienced the accident and damage had higher tolerance and positive attitudes towards the Asian elephants. Those who had seen Asian elephants in the wild had different attitudes in comparison to those who had not ever seen an elephant, and those who had seen Asian elephants had a more negative attitude.

#### 3.2.2. Villagers’ Perception of Population Change of Asian Elephants

Most of the villagers (*n* = 147, 67.7%) believed that the population of Asian elephants had increased in the past 10 years. Some villagers (*n* = 55, 25.3%) said that the population of Asian elephants had not changed significantly in the past ten years. A few villagers (*n* = 15, 6.9%) thought that the population of Asian elephants had decreased in the past 10 years. In interviews, villagers revealed that they based their opinions on the following evidence: (1) the frequency of finding Asian elephant traces during production, such as footprints, lying tracks, and food traces; (2) the frequency of Asian elephant accidents; and (3) the number of warnings (elephant approach) issued by nature reserves. The villagers believed that the main reason for the increase in the Asian elephant population was the work of Xishuangbanna Nature Reserve and the improvement of residents’ awareness of wildlife protection.

#### 3.2.3. Villagers’ Selection Preference for Mitigating Human–Elephant Conflict

**Figure 3:**
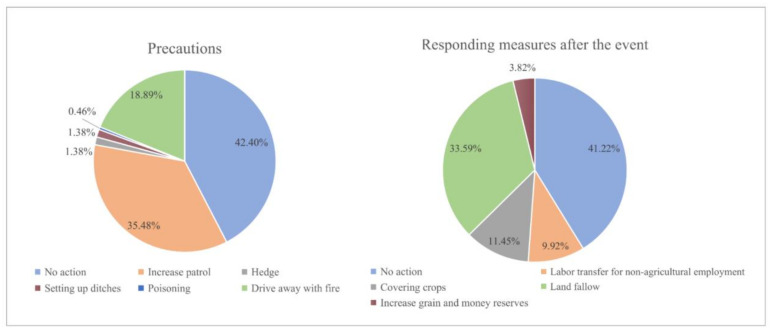
Villagers’ selection preference for mitigating human–elephant conflicts in Xishuangbanna Nature Reserve.

Most of the villagers assumed a passive role in preventing the human–elephant conflict and after elephant accidents (Figure 3). Most of the villagers did not take any measures (precaution: *n* = 92, 42.40%; after the event, *n* = 54, 41.22%). In the selection preference of preventive measures, most people chose to increase patrol (*n* = 77, 35.48%), followed by driving away elephants with fire (*n* = 41, 18.89%). Only a small number of people, accounting for about 1% of the total, chose to set a hedge, ditches, or use poison. After the elephant accident, most people chose fallow (*n* = 44, 33.59%), followed by covering crops (*n* = 15, 11.45%) and labor non-agricultural employment transfer (*n* = 13, 9.92%), and few people chose to increase food and money reserves (*n* = 5, 3.82%).

## 4. Discussion

### 4.1. Characteristics of Human–Elephant Conflict

More than 60% of the villagers in this study suffered different types of accidents associated with the Asian elephants. The Xishuangbanna Nature Reserve faces a serious problem of human–elephant conflict. The total number of Asian elephants in China is approximately 184–205 [13] and most of these elephants are found in Xishuangbanna National Nature Reserve, with a few also in Pu’er city and Nangunhe National Nature Reserve. However, the Asian elephant damage accounts for more than 85% of the wildlife damage in Xishuangbanna, and the highest is 98.7%. The specific objects damaged by the Asian elephants are people, livestock, food crops, and cash crops in mountainous areas, mid-mountainous areas, and around the National Nature Reserve. The loss of food crops and cash crops accounts for the highest proportion of total losses, with an average of more than 90% and the most destroyed crops are rice and corn among food crops, and rubber and sugarcane among cash crops. These four kinds of crops are the main food sources of the Asian elephants [40]. The food source of the Asian elephants is greatly limited due to its poor habitat, which leads to elephants turning to farmland for food. This feature is also reflected in the time scale of the Asian elephants’ accidents. Due to the relatively superior local natural conditions, agricultural production can be carried out throughout the year, and crops can be planted in all seasons [41]. Therefore, the human–elephant conflict occurs throughout the whole year, but the high incidence period is from April to November, which is the sowing and maturation period of the main crops.

It is worth noting that although the conflict between humans and elephants has affected the lives of local residents, most of the residents still have a positive attitude towards the elephants and do not want the number of the Asian elephants to decrease. This may be closely related to the local historical cultural tradition and current ecological and environmental protection education. For a long time, Xishuangbanna has been deeply influenced by Southern Buddhism, and the white elephant has a special significance in Southern Buddhism. White elephants are regarded as sacred animals, representing auspicious signs, and cannot be harmed [42]. Even in various large religious groups, white elephants or ordinary elephants participate in worship activities. This makes the Asian elephant more totemic to the local residents, which gives them spiritual sustenance [43]. Currently, almost all the Asian elephants in China are found in Xishuangbanna [27], especially in and around the nature reserve, which makes the Asian elephant and Xishuangbanna closely bound together. The Asian elephant has become an animal and cultural symbol in Xishuangbanna [44], which stimulates the pride of the local people and deepens the villagers’ love for Asian elephants. They think that protecting the Asian elephant is protecting certain characteristics of the local cultural heritage and the unique tropical rainforest environment. In addition, it has something to do with the publicity of ecological protection. Almost all our respondents knew that Asian elephants are national protected animals, which is important for the villagers’ positive attitude towards the Asian elephants. In the process of ecological protection education, publicity based on ecological knowledge and combined with local traditional culture and customs make people more receptive and tolerant of wildlife and conscious of wildlife protection.

The results showed that male respondents, those engaged in agricultural production, those who had experienced crop damage, and those who had seen Asian elephants were more likely to have negative attitudes toward Asian elephants. This may be affected by the social-ecosystem relationship between the local community and the Asian elephants. The main type of Asian elephant accident is the damage of food crops and cash crops. Rice, banana, and rubber are the main economic sources for local villagers. Asian elephant damage directly affects the livelihood of the villagers. Therefore, those who are engaged in agricultural production and have experienced crop damage are more sensitive to the Asian elephant accidents and have a more negative attitude. Males are the main labor force in agricultural production, so males tend to show more negative attitudes. If the villagers have not seen Asian elephants, their impressions of Asian elephants often remain in beautiful symbolism. After seeing Asian elephants in person, the villagers’ attitudes will change due to their actual feelings. However, negative attitudes are not conducive for the protection of Asian elephants [45]. Therefore, it is necessary to strengthen the education of these groups and actively encourage them to participate in the biodiversity conservation of nature reserves, such as joining the ecological patrol team, to enhance their protection awareness and tolerance of wildlife accidents. In addition, the compensation mechanism for wildlife accidents is also an effective way to enhance villagers’ tolerance to Asian elephants. The compensation mechanism does not only improve people’s tolerance for the animals causing accidents, but also minimizes the economic losses caused by the continuous wildlife damage [46,47]. Therefore, a sound compensation mechanism for wildlife accidents can play an important role in protecting wildlife and improving people’s livelihood [48].

Due to the long-term coexistence of local residents and animals, and their understanding of the historical development of animals and the factors affecting population change, their cognition of the trend of species population change is reasonable [49]. Most respondents in this study believed that the population of the Asian elephant in Xishuangbanna Nature Reserve has increased in the past 10 years, which is mainly due to the work of Xishuangbanna Autonomous Prefecture Government and Xishuangbanna Nature Reserve, and the improvement of wildlife protection awareness among local residents. Since 1998, Xishuangbanna has promulgated a law banning guns and hunting, and severely punished the hunting of endangered wild animals such as Asian elephants. Therefore, there is no poaching of Asian elephants in Xishuangbanna. Coupled with the multidirectional protection and publicity of Xishuangbanna Nature Reserve, the number of elephants in Xishuangbanna has been increasing in the past 40 years, from 101 in 1976 to 136–179 in 2006 to 184–205 in 2016 [27]. The increase in the Asian elephant population may increase the frequency of accidents, but the real reason is the change in the relationship between humans and elephants [50].

### 4.2. Causes of Human–Elephant Conflict

First, the rapid growth of the population is the root cause of the human–elephant conflict. Sun Gang’s research in 1998 shows that 20 people/km^2^ is the maximum population pressure threshold that Asian elephants can bear [51]. However, the population of Xishuangbanna exceeded 1,180,000 by 2017, and the population density is about 60 people/km^2^, which is far beyond the tolerable threshold of Asian elephant survival, which lays the tone for the occurrence of human–elephant conflict. Along with rapid population growth, the development of agriculture, tourism, and the construction of roads and cities have led to the loss of the habitat of Asian elephants, which is the main fuse of human–elephant conflicts. With the vigorous development of economic crops (tea, rubber, etc.), the original virgin forest has been completely and permanently removed, and it is no longer the food source of herbivores such as Asian elephants, and the habitat is more fragmented [52], resulting in a serious shortage of natural food and a reduction in habitat area. Asian elephants are large terrestrial herbivores. A single elephant needs 135–300 kg of food per day to meet its living conditions [53,54]. To meet the required food consumption, each wild elephant has a living area of tens of square kilometers [55]. Therefore, there must be enough habitat and food to satisfy the survival of Asian elephants. In recent years, due to the rising market prices of rubber, tea, and other cash crops, local villagers have vigorously developed plantations, resulting in serious destruction of forest resources and rapid reduction of forest area. The area where rubber is suitable for planting is generally less than 1000 m above sea level [56], and this area is usually the most suitable living place for Asian elephants [57]. A large number of reclamation and cultivation have led to the continuous decrease in the habitat and food sources for Asian elephants, thus forcing the Asian elephants to go out of the forest to seek food in the surrounding agricultural land, forming a situation in which humans and elephants compete against each other for living space.

Second, with the change in the relationship between human and elephants, the change in living habits of Asian elephants also aggravates the conflict between humans and elephants. With the destruction of the living environment, under the condition of a limited range of activities and serious shortage of food in the forest, the Asian elephant must go out of the forest for food [58]. Asian elephants have a new demand for food, which has changed their previous feeding behavior. Asian elephants prefer sugarcane, corn, banana, and other crops to bamboo and other wild plants. These foods are relatively concentrated and easy to eat [55], and food from crops has replaced food from wild plants. In recent years, Asian elephants have gradually understood the maturity cycle of crops. Once the crops are ripe, they swarm into crop areas to feed on crops, thus aggravating the conflict with the local villagers.

Finally, there is the animosity of Asian elephants towards human beings. In the maturation season, Asian elephants enter the crop area to eat and damage crops, which seriously affects the production and life of local villagers. To safeguard their crops, the villagers have adopted many ways to drive away the Asian elephants, such as roaring, setting fire, beating gongs, and drums. Long-term exposure to human hostility may make elephants both over-reactive and hostile to people in response. They take a series of revenge actions against human beings, such as destroying production tools and workshops. When they meet people at close range, they often take the initiative to attack human beings. In recent years, Asian elephant injuries have occurred almost every year.

### 4.3. Problems in Compensation for Human–Elephant Conflict

After the Xishuangbanna government included the wildlife damage accidents into the scope of public liability insurance, there has been great progress in compensation for the Asian elephant damage [59], but there are still some deficiencies in the process of insurance compensation.

First, the workload for field inspectors is too much. At present, there are about 150 workers in the field to assess the damage, including insurance company personnel, staff of the Protection Institute and forestry station, long-term forest protection personnel, and village cadres. However, the damage from the human–elephant conflict is wide-ranging and covers a large area. For example, in 2012, 2013, 2014, and 2017, the number of villages experiencing wildlife accidents in the reserve exceeded 50% of the total number of villages in and around the reserve. Moreover, most of the areas with a high incidence of wildlife accidents are mountainous and other areas that are difficult to reach, which result in a heavy workload for field inspection. This also makes it difficult for field inspectors to determine the loss and establish sufficient basis for claim settlement.

Second, qualitative and quantitative analyses of damage are difficult. The policy for wildlife accident compensation is strong, which requires an accurate grasp of the compensation scope and compensation object. The economic losses associated with human–elephant conflict mainly include food, cash crops, property loss, and personal injury. It is difficult to determine the quality of inspection standards for grain and cash crops, and the time during which the drops are damaged directly affects the output. For example, some crops are damaged after replanting or a piece of land has been repeatedly damaged, and this affects the output.

Finally, the determination of compensation standards lacks a scientific basis. The valuation of loss for wildlife compensation is based on the market value for crop and it fluctuates depending on the market price. However, due to the lack of a standard basis for value evaluation and failure to consider the output of different land types and the environmental factors affecting crop growth, a unified compensation standard has been adopted, which results in low compensation for land types with high output.

To sum up, as a result of these problems and the time required to process the claims, some victims give up reporting wildlife incidents. However, the low reporting rate is not conducive for the protection of wildlife [60,61]. Therefore, it is necessary to efficiently collect the evidence of human–elephant conflict, simplify the procedures of claims settlement, increase the amount of compensation, and shorten the period of compensation for effective compensation in wildlife accidents.

### 4.4. Countermeasures of Human–Elephant Conflict Mitigation

The government of Xishuangbanna Autonomous Prefecture and Xishuangbanna Nature Reserve have taken several preventive and management measures to reduce the Asian elephant damage. For example, the establishment of an Asian elephant food source base and the construction of an electric fence, elephant proof ditch, and other facilities [31]; rural energy construction; call on residents to adjust the planting structure; adjust short-term cash crops to long-term economic trees; and develop the courtyard planting industry dominated by *Dendrobium* (Asian elephants do not like to eat this kind of plant, and the market value is high); improve the multiple cropping index of agricultural crops and increase the yield. These measures have played a certain role in reducing and preventing the Asian elephant damage, but have not significantly reduced the incidence of human–elephant conflicts. Presently, most villagers take passive measures to deal with human–elephant conflicts, and it is worth noting that a considerable number of villagers have chosen to leave fallow their agricultural land and seek non-agricultural employment after suffering from elephant accidents. This not only shows the extent of the impact of human–elephant conflict on residents, but also shows that in the face of such conflicts, local villagers can take few effective measures. Villagers are pushed into a precarious position, with their livelihood, property, and life in danger. The forced change in livelihood style as a result of the human–elephant conflict may instigate negative attitudes among villagers toward the Asian elephants, thus jeopardizing the effectiveness of Asian elephant protection. Therefore, in any initiatives for the protection of Asian elephants, it is important to consider the living conditions of local residents and ensure that they have a positive attitude towards the Asian elephants. In addition, joint effort from the government, society, and local villagers is required to ensure the protection of the Asian elephant population, reduction of loss of life and property for residents, and effective alleviation of the problem of human–elephant conflict.

What frequently instigates the human–elephant conflict is the encroachment of human living space into elephant habitat. Conflict between human beings and Asian elephants is inevitable when both compete for limited natural resources and land. Therefore, only by separating the Asian elephants from the human beings can the conflict be completely resolved. In the semistructured interviews, more than 80% of the key informants mentioned that "if we try to solve the problem of human elephant conflict thoroughly, we have to move people or Asian elephants". However, the reality is that the local government cannot solve a series of social problems brought about by the overall relocation of many villages, such as employment, housing land, distribution of means of production, and the villagers themselves do not want to leave the land where they have lived for generations. On the other hand, the suitable living environment for Asian elephants in China is narrow, so it is impossible to realize the migration of Asian elephants. Therefore, in order to alleviate the human–elephant conflict in future, it is necessary to strike a balance between the needs of Asian elephants and the needs of the human society.

The main reason for the Asian elephants invading the human living area is the declining quality of their habitat. Therefore, it is necessary to improve forest protection, further enhancing villagers’ awareness of forest resources protection, and use scientific means to restore the quality of the Asian elephants’ habitat and increase the surface area of the habitat. At the same time, due to habitat fragmentation, Asian elephants will inevitably cause losses when they pass through residential areas in the process of migration. Wildlife corridors should be built to connect the fragmented and isolated habitats of Asian elephants in order to avoid the damage associated with the migration process of Asian elephants. Finally, through the construction of food source bases, Asian elephants can be attracted to the food source bases in the maturation season of crops to reduce crop losses in surrounding villages.

At the other end of the conflict, as villagers suffer economic losses due to the impact of the Asian elephants, which reduces their willingness to protect the Asian elephants, the conflict between humans and elephants intensifies. Therefore, to reduce this conflict, villagers need to develop alternative planting measures, encourage the transfer of non-agricultural employment, and reduce their dependence on agricultural production as much as possible. At the same time, the standard and process of claim settlement need to be further improved to ensure villagers receive compensation that matches the market price, covers the economic losses associated with the Asian elephants as much as possible, and improves the villagers’ tolerance and protection enthusiasm. Moreover, by laying an infrared trigger camera alarm system and setting up an iron fence and beehive fence [62,63] around the village, a modern early warning system and defense system should be established to ensure the safety of local villagers.

## 5. Conclusions

Taking the example of Xishuangbanna Nature Reserve, where Asian elephants are most concentrated in China, this study analyzed the types, causes, compensation, attitudes, and cognition of human–elephant conflicts, and puts forward possible mitigation measures. With the continuous development of human society, the living environment of Asian elephants is still changing, the space struggle and conflict between Asian elephants and human beings will continue, and the relationship between local people and the Asian elephants will also change. More efforts should be made to solve this conflict in the future. In order to fundamentally reduce the intensity and frequency of human–elephant conflict and promote the coexistence of human beings and Asian elephants, researchers, and management decision makers need to strengthen the research on the relationship between human social development and the survival of Asian elephants, explore the relationship between the changes in residents’ production and lifestyle and the change in Asian elephant behavior, and strengthen the study of Asian elephant ecology, the driving factors of Asian elephant accidents in terms of habitat quality, population dynamics, natural food sources, and ecosystem integrity.

## Figures and Tables

**Table 1:** Compensation standard of public liability insurance for wildlife accidents from 2011 to 2018.

Type of Crops Planted	Compensation Standard (¥)		
	2011	2012	2013–2018
Rice/Mu	500	500	500
Rice (on the hillside)/Mu	/	/	400
Corn/Mu	400	400	400
Buckwheat/Mu	400	400	400
Soybean/Mu	250	250	250
Peanut/Mu	400	400	400
Wax gourd/Mu	/	/	300
Watermelon/Mu	/	/	500
Sugarcane/Mu	700	700	700
Rubber/Plant	10 (<5 years)	10 (<5 years)	15
	20 (≥5 years)	20 (≥5 years)	
Agilawood, Nut/Plant	2	2	10
Tea/Plant	5	5	2
Coffee/Plant	10	10	5
Banana/Plant	5	5	10
Pine/Plant	5	5	10
Fruit tree/Plant	10	10	20

Note: The limit of compensation for casualties is 200,000 yuan, including 200,000 yuan for one-time death, 200,000 yuan for disability, and 100,000 yuan for medical expenses

**Table 2:** Profiles of the respondents of questionnaire survey, *n* = 217.

Variable and Description	Frequency	Percentage (%)
**Gender in%**		
Female	40	18.43%
Male	177	81.57%
**Age in%**		
<35 Years	43	19.82%
35—55 Years	155	71.43%
>55 Years	19	8.76%
**Education level**		
<Primary school	52	23.96%
≥Primary school	165	76.04%
**Form of Occupation**		
Farmer	157	72.35%
Other	60	27.65%
**Residence (township)**		
Dadugang	12	5.53%
Shangyong	19	8.76%
Guanlei	17	7.83%
Jinuo	13	5.99%
Jingha	14	6.45%
Mengban	26	11.98%
Menghai	37	17.05%
Mengla	35	16.13%
Mangman	23	10.60%
Mengzhe	21	9.68%

**Table 3:** Comparison of compensation standards before and after implementation of public liability insurance for wildlife.

Compensation Content	Compensation Standard (¥)		Rate of Rise
	2008	2013	
Human killed	80,000	200,000	150%
Human injured	Calculated according to the actual expenses incurred		/
Rice/Mu	12	500	317%
Corn/Mu	75	400	433%
Soybean/Mu	75	250	233%
Peanut/Mu	75	400	433%
Sugarcane/Mu	105	700	567%
Rubber/Plant	2	15	650%
Tea/Plant	0.4	2	400%
Fruit tree/Plant	10	20	100%
Pine/Plant	0.5	2	300%
Coffee/Plant	0.5	5	900%
Banana/Plant	1	10	900%

**Table 4:** Difference of villagers’ attitude towards an elephant in different demographic features.

Variables	Categories	Number of Likes for Elephants	Number of Dislikes for Elephants	χ^2^	*p*
Gender	Male	106	71	7.246	0.007 **
	Female	33	7	-	-
Age	<35 years	30	13	0.762	0.683
	35–55 years	97	58	-	-
	>55 years	12	7	-	-
Education level	<Primary school	32	20	0.188	0.664
	≥Primary school	107	58	-	-
Occupation	Farmer	92	65	7.343	0.007 **
	Other	47	13	-	-
The crops have beeneaten by elephants	Yes	68	63	21.182	0.000 **
	No	71	15	-	-
Livestock attacked experience	Yes	8	6	2.921	0.087
	No	133	70	-	-
Attacked by elephants	Yes	3	2	0.037	0.848
	No	136	76	-	-
Seen elephant	Seen	97	65	4.847	0.028 *
	Have not seen	42	13	-	-

** Significant at *p* < 0.01. * Significant at *p* < 0.05.
